# Salvage Reirradiation for Locally Recurrent Prostate Cancer: Results From a Prospective Study With 7.2 Years of Follow-Up

**DOI:** 10.1200/JCO.23.01391

**Published:** 2024-04-23

**Authors:** Christian Ekanger, Svein Inge Helle, Lars Reisæter, Liv Bolstad Hysing, Rune Kvåle, Alfred Honoré, Karsten Gravdal, Sara Pilskog, Olav Dahl

**Affiliations:** ^1^ Department of Oncology and Medical Physics, Haukeland University Hospital, Bergen, Norway; ^2^ Department of Radiology, Haukeland University Hospital, Bergen, Norway; ^3^ Department of Clinical Medicine, University of Bergen, Bergen, Norway; ^4^ Department of Technology and Physics, Faculty of Mathematics and Natural Sciences, University of Bergen, Bergen, Norway; ^5^ Department of Research, Cancer Registry of Norway, Oslo, Norway; ^6^ Department of Urology, Haukeland University Hospital, Bergen, Norway; ^7^ Department of Patohology, Haukeland University Hospital, Bergen, Norway; ^8^ Department of Clinical Science, University of Bergen, Bergen, Norway

## Abstract

**PURPOSE:**

There are no well-established re-treatment options for local recurrence after primary curative radiation therapy for prostate cancer (PCa), as prospective studies with long-term follow-up are lacking. Here, we present results from a prospective study on focal salvage reirradiation with external-beam radiation therapy with a median follow-up of 7.2 years.

**MATERIALS AND METHODS:**

From 2013 to 2017, 38 patients with biopsy-proven locally recurrent PCa >2 years after previous treatment and absence of grade 2-3 toxicity from the first course of radiation were included. The treatment was 35 Gy in five fractions to the MRI-based target volume and 6 months of androgen-deprivation therapy starting 3 months before radiation. The Phoenix criteria defined biochemical recurrence-free survival (bRFS), and toxicity was scored according to Radiation Therapy Oncology Group criteria.

**RESULTS:**

Median age was 70 years, and median time from primary radiation to prostate-specific antigen (PSA) recurrence was 83 months. The actuarial 2-year and 5-year bRFS were 81% (95% CI, 69 to 94) and 58% (95% CI, 49 to 74), respectively. The actuarial 5-year local recurrence-free survival was 93% (95% CI, 82 to 100), metastasis-free survival was 82% (95% CI, 69 to 95), and overall survival was 87% (95% CI, 76 to 98). Two patients (5%) had durable grade 3 genitourinary toxicity, one combined with GI grade 3 toxicity. A PSA doubling time ≤6 months at salvage, a Gleason score >7, and a PSA nadir ≥0.1 ng/mL predicted a worse outcome.

**CONCLUSION:**

Reirradiation with EBRT for locally recurrent PCa after primary curative radiation therapy is clinically feasible and demonstrated a favorable outcome with acceptable toxicity in this prospective study with long-term follow-up.

**Figure:**
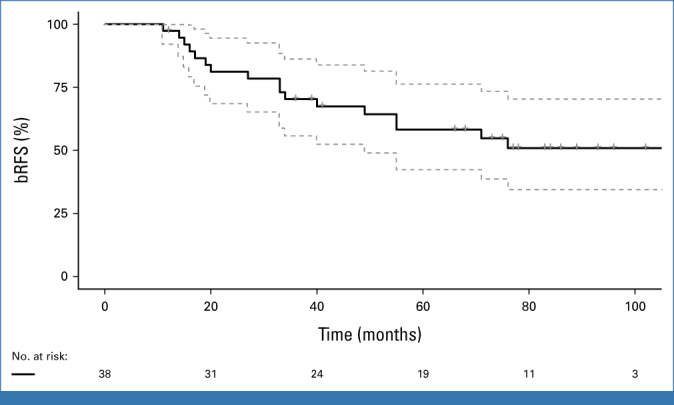

## INTRODUCTION

Prostate cancer (PCa) is the second most commonly diagnosed cancer and the fifth leading cause of cancer-related death among men worldwide.^1^ External-beam radiation therapy (EBRT), with or without androgen-deprivation therapy (ADT), is a curative treatment option for localized and locally advanced cancers and can be offered to patients in all prognostic risk groups.^2^ However, despite advances in EBRT delivery techniques and dose-escalated radiation, up to 15% of patients develop local recurrence within 10 years after treatment.^3,4^ Local recurrence is a risk factor for further disease progression and death, especially in patients with high-risk disease at initial treatment.^5^

**CONTEXT**

**Key Objective**
Is reirradiation with external-beam radiation therapy (EBRT) administered as 7 Gy × five combined with short-term androgen-deprivation therapy an effective and feasible curative treatment option for patients with a local recurrence after primary radiation therapy for prostate cancer?
**Knowledge Generated**
Five-year biochemical recurrence-free survival after reirradiation with EBRT was 58% in this prospective study on 38 patients with a median follow-up of 7.2 years. Two patients (5%) experienced durable grade 3 toxicity; one underwent a cystoprostatectomy with rectal amputation.
**Relevance *(J.P.S. Knisely)***
The optimal approach for salvaging local recurrences after definitive prostate radiation is unknown. Reirradiation with a hypofractionated regimen has been tested and may be an approach that merits formal randomized testing against other focal therapies such as focused ultrasound or cryotherapy.*
  *Relevance section written by *JCO* Associate Editor Jonathan P.S. Knisely, MD.

Unlike recurrence after radical prostatectomy (RP), there are no well-established treatment options for local recurrence after primary curative radiation therapy. There is a lack of high-quality data showing the beneficial effects of re-treatment on survival, and there is an increased risk of complications. Consequently, only 2%-3% of patients are offered curative-intent re-treatment.^6,7^ Most patients are treated with life-long ADT, a palliative treatment strategy with significant negative impacts on quality of life because of a wide range of side effects.^8,9^

Consensus recommendations on salvage options for locally recurrent nonmetastatic disease are limited. According to the European Association of Urology (EAU) guidelines, salvage RP can be offered to highly selected patients.^10^ However, it is technically demanding and associated with a substantially higher risk of complications than primary RP.^10-13^ Other therapies, such as high-intensity focused ultrasound (HIFU), cryosurgical ablation, and salvage brachytherapy, can be offered to patients with proven local recurrence within a clinical trial setting or a well-designed prospective cohort study. Reirradiation with EBRT is not mentioned as an alternative in the EAU guidelines.^10^ The American Society for Radiation Oncology and National Comprehensive Cancer Network clinical practice guidelines do not comment on reirradiation with EBRT.^14,15^

A systematic review of salvage RP for radiorecurrent PCa from 2021 reported 5-year biochemical recurrence-free survival (bRFS) ranging from 34% to 83% and 10-year bRFS ranging from 31% to 37%.^16^ All the studies included in the analyses had a retrospective design. In two updated systematic reviews and a meta-analysis of local salvage therapies after radiotherapy for PCa, only two prospective studies on reirradiation with EBRT were found, with 25 months and 44 months of follow-up, respectively.^17-20^ Actuarial 2-year bRFS rates in these two studies were 80% and 76%, respectively, and the 5-year bRFS was 60%.

This prospective study aimed to evaluate the effect and feasibility of reirradiation with EBRT in 38 patients with local recurrence after primary radiation therapy, with a median follow-up of 7.2 years.

## MATERIALS AND METHODS

### Patients

Patients eligible for the study had to meet the following inclusion criteria: (1) biopsy-proven locally recurrent adenocarcinoma in the prostate gland at least 2 years after the primary curative intended radiation therapy, (2) no spread of disease beyond the periprostatic tissue, (3) absence of greater than Radiation Therapy Oncology Group (RTOG) grade 1 toxicity from the primary radiation course, and (4) a life expectancy longer than 5 years.

All patients underwent multiparametric magnetic resonance imaging (mpMRI) of the pelvis before ADT was initiated as the basis for treatment planning. No exclusion criteria were derived from mpMRI concerning tumor size or location. A bone scan was performed if the prostate-specific antigen (PSA) was >10 ng/mL. Five patients underwent 18F-fluciclovine (18F-FACBC) positron emission tomography-computed tomography (PET-CT) before reirradiation. Patients with distant or lymph node metastases on imaging were excluded from the study. Treatment started with ADT 3 months before reirradiation for a total duration of 6 months (Table 1). The clinician decided on the imaging modality when biochemical recurrence occurred after re-treatment.

**TABLE 1.** Patient Characteristics at Initial Diagnosis and at Recurrence

Patient Characteristic	Number
No. treated	38
Age at salvage radiation, years, median (range)	70 (59-84)
Primary radiation dose (EQD2 a/b, 1.5) Gy, median (range)	78 (66-81)
Primary EBRT dose ≤70 Gy, No. (%)	10 (26.3)
Primary dose-escalated EBRT >70 Gy, No. (%)	28 (73.7)
Primary EBRT given in standard 2 Gy fractions, No. (%)	20 (52.6)
Primary hypofractionated EBRT (>2 Gy), No. (%)	
2.15 Gy × 35	4 (10.5)
2.7 Gy × 25	14 (36.8)
Time from primary radiation to PSA recurrence, months, median (range)	83 (30-158)
Risk group at initial diagnosis, No. (%)	
Low	6 (15.8)
Intermediate	8 (21.1)
High	23 (60.5)
Not known	1 (2.6)
Gleason score at initial diagnosis, No. (%)	
Gleason score 6	15 (39.5)
Gleason score 7a	9 (23.7)
Gleason score 7b	6 (15.8)
Gleason score 8	7 (18.4)
Gleason score 9	1 (2.6)
T stage (mainly DRE) at initial diagnosis, No. (%)	
T1	12 (31.6)
T2	9 (23.7)
T3	16 (42.1)
Not known	1 (2.6)
PSA levels, median 15 (5.3-75) at initial diagnosis, ng/mL, No. (%)	
PSA<10	12 (31.6)
PSA 10-20	13 (34.2)
PSA>20	12 (31.6)
Not known	1 (2.6)
Gleason score at salvage, No. (%)	
3 + 3	1 (2.6)
3 + 4 = 7a	10 (26.3)
4 + 3 = 7b	15 (39.5)
4 + 4 = 8	8 (21.1)
4 + 5 = 9a	3 (7.9)
5 + 4 = 9b	1 (2.6)
T stage (DRE/mpMRI) at salvage, No. (%)	
T1	19 (50)
T2	15 (39.5)
T3	1 (2.6)
Not classified	3 (7.9)
PSA levels, ng/mL, median (range), at salvage	4 (2-23)
PSA-DT at salvage, months, No. (%)	
<6	7 (18.4)
6-12	13 (34.2)
>12	18 (47.4)
Treatment volume, cm^3^, median (range)	32.75 (11.3-90.5)
Prostate coverage from focal reirradiation, No. (%)	
One lobe	13 (34.2)
Both lobes	17 (44.7)
Whole gland	8 (21.1)
ADT for 6 months, No. (%)	
LHRH analog	13 (34.2)
Antiandrogen	24 (63.2)
None	1 (2.6)
Follow-up overall survival, months, median (range)	96 (15-120)
Follow-up PSA to recurrence or death, months, median (range)	55 (11-111)
Follow-up PSA patients alive and recurrence-free, months, median (range)	88 (66-111)

Abbreviations: a/b, alpha/beta ratio; ADT, androgen-deprivation therapy; DRE, digital rectal exploration; DT, doubling time; EBRT, external-beam radiotherapy; EQD2, equivalent dose in 2 Gy fractions; LHRH, luteinizing hormone-releasing hormone; mpMRI, multiparametric magnetic resonance imaging; PSA, prostate-specific antigen.

### Planning and Treatment Technique

The prescribed dose was 7 Gy × five fractions for a total dose of 35 Gy, delivered as one fraction per day and three fractions per week (every other day). All patients had fiducial gold markers implanted in the prostate before planning CT acquisition. Gross tumor volume (GTV) was defined on CT as areas suspicious for cancer within the prostate or seminal vesicles on the basis of coregistered mpMRI. Subsequently, this GTV was expanded to form a clinical target volume (CTV) considering the GTV and the extent of positive biopsies. The biopsy process was not prespecified and varied between standard (10-12 cores) and targeted biopsies.^21^ A 5-mm planning target volume (PTV) margin was added around the CTV in all directions except toward the rectum. GI toxicity was limited by constraining the maximum rectal dose to 25 Gy (equivalent dose in 2 Gy fractions [EQD2]). No specific constraints were applied for the urethra or the bladder. Treatment was delivered using volumetric-modulated arc therapy or intensity-modulated radiotherapy. PTV coverage (D98%) was between 95% and 107% of the prescribed dose.

### End Points

According to the protocol, the primary end points were clinical and biochemical disease control, including bRFS, local control, and distant metastasis-free survival (MFS).^22,23^ bRFS was defined according to the Phoenix definition (PSA rise 2 ng/mL after nadir).^24^ Secondary end points encompassed peak RTOG acute and late genitourinary (GU) tract and GI toxicity.^25^ Overall survival (OS) estimates were performed as post hoc analysis. Additionally, we evaluated whether the Gleason score, PSA level, and PSA doubling time (PSA-DT) at salvage could predict the risk of recurrence after re-treatment. We also assessed if a PSA nadir after re-treatment that was ≥0.1 ng/mL influenced recurrence rates. Death due to PCa was defined as death during the metastatic castration-resistant phase.

### Statistical Methods

The initially approved sample size was 20 patients, with a stop of the trial if four acute grade 3 toxicities appeared. Only one acute and two late grade 3 toxicity were observed, and the PSA declined to undetectable levels in 15 of the 20 first patients. Consequently, considering the observed response rate of 75%, a confidence level of 85%, and a margin error set at 10%, a sample size of 39 patients was calculated in the subsequent phase, and an expansion to 40 patients was approved. Statistical analyses were performed using SPSS (version 29.0; IBM Corp, Armonk, NY). The reverse Kaplan-Meier method was used to estimate the median follow-up time.^26^ Kaplan-Meier estimates were calculated to assess outcomes for bRFS, freedom from local recurrence, MFS, and OS, and the Cox proportional hazard model was used to assess the association between bRFS and predictor variables. We did not perform multivariable analyses because of the small number of patients. Curves were formed using R Development Core Team version 4.2.2.

### Ethics

The Regional Ethical Committee in Western Norway approved the study protocol (REK no. 25228; 2012/1868), and all patients provided written informed consent for participation.

### Follow-Up

Follow-up assessments were performed on the last day of treatment. Acute toxicity was scored using the RTOG criteria on the last day of treatment and at 3 months. After that, follow-up assessments were scheduled every third month up to 12 months and subsequently every 6 months.

## RESULTS

Between March 2013 and April 2017, 38 consecutive patients were included in the study. The median follow-up was 86 months (range, 11-111 months), starting from the initiation of ADT until the last recorded control with measurement of PSA. Median follow-up on OS was 96 months (range 15-120). The median age was 70 years (range, 59-84 years), and the median primary radiation dose was 78 Gy (range, 66-81 Gy) EQD2 (alpha/beta ratio, 1.5). All patients received primary radiation therapy as EBRT, 20 patients with a 2 Gy fraction schedule and 18 with hypofractionation (2.15 Gy × 35 or 2.7 Gy × 25). Twenty-three patients (60%) were classified as high risk in the primary situation. The median interval from primary treatment to PSA recurrence was 83 months (range, 30-158 months). Thirteen patients received focal reirradiation limited to one half of the prostate. In 17 patients, the PTV extended into the contralateral half, and in eight, the PTV included the whole gland. All patients received radiation therapy every other day for 10 days, except for one who once received radiation on 2 consecutive days. One patient did not receive ADT because of high age. Patient characteristics are shown in Table 1.

**FIG 1.**
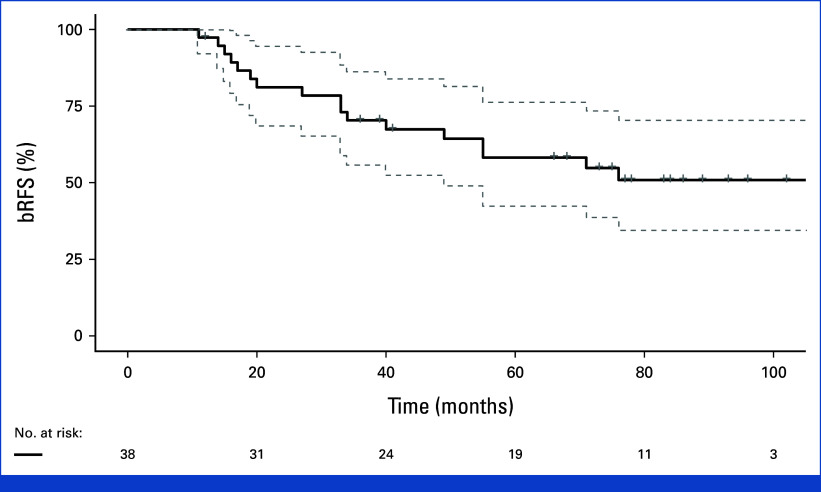
Treatment outcome. Kaplan-Meier plot of bRFS in the whole patient group with 95% CI. bRFS, biochemical recurrence-free survival.

The actuarial 2-year and 5-year bRFS rates were 81% (95% CI, 69 to 94) and 58% (95% CI, 49 to 74), respectively (Fig 1). The actuarial 5-year local recurrence-free survival was 93% (95% CI, 82 to 100), MFS was 82% (95% CI, 69 to 95), and OS was 87% (95% CI, 76 to 98). One patient (3%) died of PCa 86 months after treatment, and four others (11%) reached the metastatic castration-resistant phase. Twelve of 17 recurrence patients (71%) were treated with palliative ADT. Two patients died during follow-up from infections without ADT and no signs of clinical progression or metastasis. One patient had new curative-intent radiation against pelvic lymph nodes and had no PSA rise during 4 years of follow-up after the second recurrence. Two patients with a marginal PSA rise were observed with stable disease without treatment. Sixteen patients (42%) were alive without recurrence at the end of the study.

**FIG 2.**
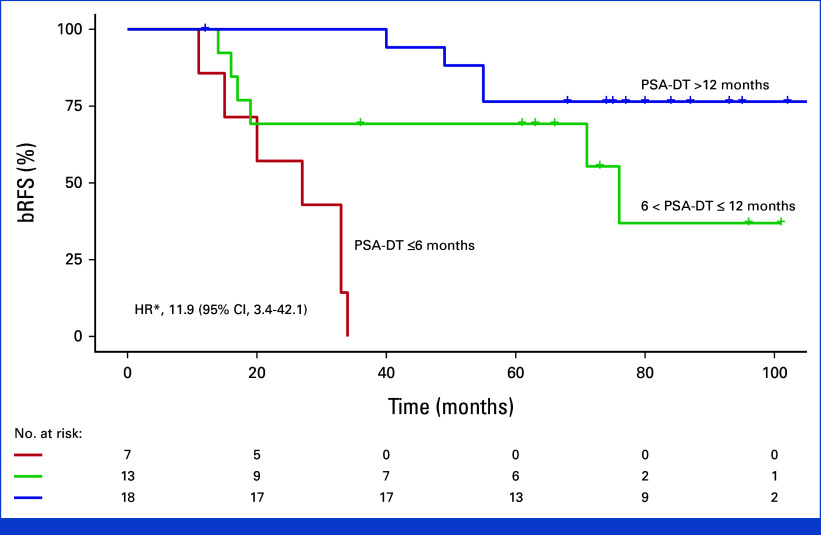
Impact of PSA doubling time at salvage on treatment outcome. Kaplan-Meier plot of bRFS. HR*, 11.9 (95% CI, 3.37 to 42.1; PSA-DT ≤6 months/the two other groups); HR, 4.49 (95% CI, 1.28 to 15.8; PSA-DT ≤6 months/6 < PSA-DT ≤ 12 months). bRFS, biochemical recurrence-free survival; HR, hazard ratio; PSA-DT, prostate-specific antigen doubling time.

**FIG 3.**
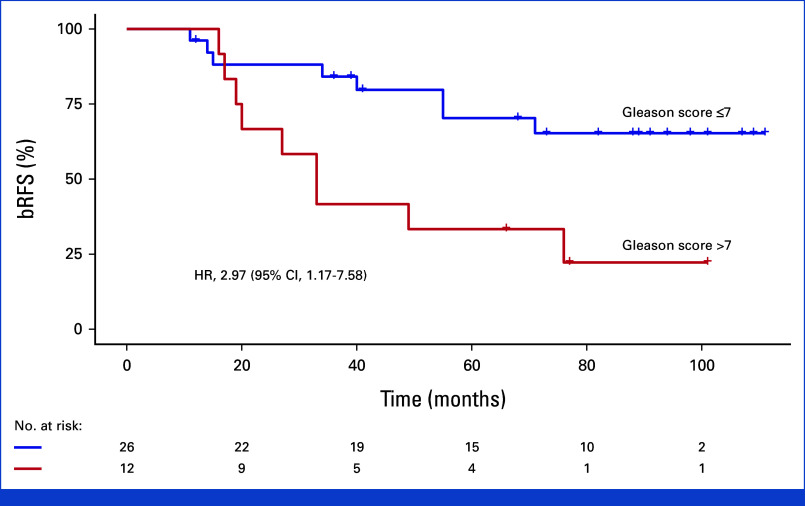
Impact of Gleason score at salvage on treatment outcome. Kaplan-Meier plot of bRFS. bRFS, biochemical recurrence-free survival; HR, hazard ratio.

Univariable analysis demonstrated a considerably increased recurrence risk with a short PSA-DT ≤6 months before re-treatment. Five-year bRFS was 0% versus 69% and 77% for patients with PSA-DT ≤6 months, PSA-DT 6-12 months, and PSA-DT >12 months, respectively (hazard ratio [HR], 11.9 [95% CI, 3.37 to 42.1]; Fig 2). We also observed a worse bRFS for patients with a Gleason score >7 at salvage, with a 5-year bRFS of 33% compared with 70% for patients with a Gleason score ≤7 (HR, 2.97 [95% CI, 1.17 to 7.58]; Fig 3). The Fisher-Freeman-Halton test demonstrated a significant association between the Gleason score and PSA-DT (*P* = .024). We found no association between PSA levels at salvage and the risk of recurrence.

**FIG 4.**
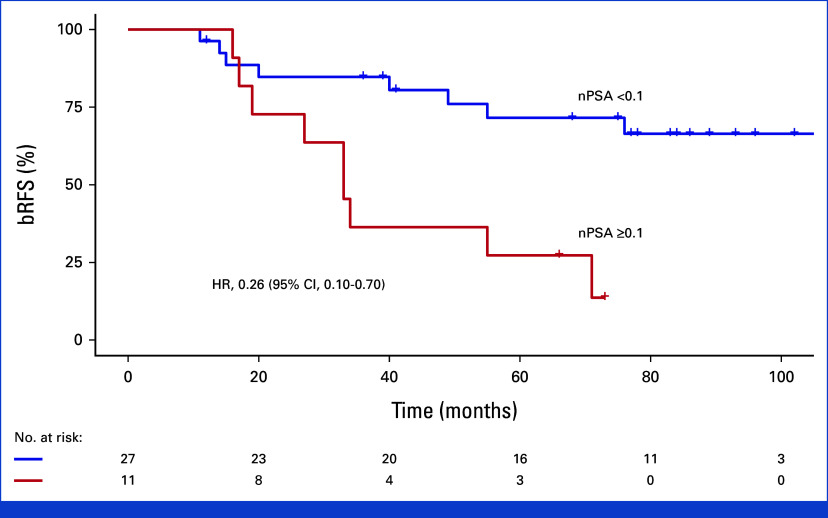
Impact on nPSA after salvage. bRFS, biochemical recurrence-free survival; HR, hazard ratio; nPSA, nadir prostate-specific antigen.

Patients reaching a PSA nadir value <0.1 ng/mL (n = 27) had a considerably better outcome than the rest (n = 11), with 5-year bRFS 72% compared with 27%, respectively (HR, 0.26 [95% CI, 0.10 to 0.70]; Fig 4). In 23 of 27 patients, the nadir value <0.1 ng/mL was achieved at the first 3-month follow-up. All four patients with a subsequent achieved nadir value <0.1 ng/mL remained recurrence-free.

Data on GU and GI toxicities were complete for all the patients. The peak acute RTOG GU and GI scores were grade 2 in 18% and 5% of patients, respectively, and acute grade 3 GU and GI toxicity were observed in 5% and 0%, respectively (Table 2). Peak late RTOG GU and GI grade 2 toxicities occurred in 26% and 8% of patients, respectively, and peak late RTOG grade 3 was observed in 16% and 3%, respectively. One patient with late grade 3 combined GU and GI toxicity required cystoprostatectomy with rectal amputation, and one with late grade 3 GU toxicity required a permanent suprapubic catheter. Other late grade 3 events resolved spontaneously to grade 2 (n = 1), grade 1 (n = 2), or grade 0 (n = 1) within 6-9 months. Five patients received hyperbaric oxygen therapy because of late toxicity. In a separate analysis of the relationship between radiation exposure and side effects in 28 patients from the study, exposure of more than 35% of the bladder to a cumulative dose higher than 50 Gy (EQD2) increased the GU late grade 2 toxicity.^27^ No relationship was found between GI toxicity and the cumulative dose.

**TABLE 2.** Peak Acute and Late RTOG Toxicity

RTOG Toxicity Grade	GU Toxicity, No. (%)		GI Toxicity, No. (%)	
	Acute	Late	Acute	Late
Grade 0	14 (37)	8 (21)	26 (68)	28 (74)
Grade 1	15 (39)	14 (37)	10 (26)	6 (16)
Grade 2	7 (18)	10 (26)	2 (5)	3 (8)
Grade 3	2 (5)	6 (16)	—	1 (3)
Grade 4	—	—	—	—

Abbreviations: GU, genitourinary; RTOG, Radiation Therapy Oncology Group.

## DISCUSSION

This study provides important information for managing patients with locally recurrent PCa initially treated with EBRT. To our knowledge, it is the third prospective study on salvage reirradiation with EBRT and the first with a follow-up beyond 5 years. The results showing a 5-year bRFS of 58% are consistent and comparable with the other prospective long-term follow-up study by Fuller et al,^17^ reporting a 5-year bRFS of 60%. That study used a similar fractionation schedule of 34 Gy administered in five consecutive daily fractions of 6.8 Gy, but with a radiation technique not widely available (CyberKnife). Although reirradiation in this study was focal, the covered prostate volume extended into the contralateral half in most patients. The results compare favorably to studies on salvage RP, which are mainly retrospective with an estimated 5-year bRFS of 54% (95% CI, 49 to 59).^18^ Related to other nonsurgical salvage options, the results are comparable with brachytherapy (5-year bRFS of 60% using high dose rate and 56% with low dose rate) and favorable compared with HIFU and cryotherapy, with estimated 5-year bRFS rates of 53% and 50%, respectively.^18^

In patients with rising serum PSA levels after RP and radiation therapy, a short PSA-DT is an adverse prognostic predictor.^28-34^ A short PSA-DT at salvage also predicted a worse prognosis in our study; all seven patients with PSA-DT ≤6 months had recurrence. Although the sample size was small, evaluating PSA-DT at recurrence may help clinicians select patients with a curative potential.

The value of prostate biopsy after radiation therapy for PCa in predicting survival and recurrence is uncertain.^35^ In this study, a Gleason score >7 at salvage was associated with a greater risk of recurrence. The curative potential of re-treatment may be smaller in these patients. However, they may well be the ones who can benefit the most from re-treatment if tumors are controlled, as the Gleason score strongly influences the risk of disease progression in the primary setting.^36^ Our study demonstrated a significant association between the Gleason score and PSA-DT. Previous studies have established that these factors are independent predictors of outcome.^32,33,37^

A PSA nadir <0.1 is strongly prognostic of bRFS and long-term survival in patients with localized PCa treated with primary radiation therapy.^38^ This study indicates that the PSA nadir value is also a prognostic factor after salvage treatment.

We did not find an association between absolute PSA levels at salvage and recurrence risk, as shown in the study by Fuller et al.^17^ However, our study included only seven patients with a PSA >6.92 ng/mL, which emerged as the sharpest cutpoint in the study by Fuller et al.

The complication rates in this study were denoted as peak values to indicate the worst possible outcomes. Six patients (16%) experienced late grade 3 GU toxicity during the follow-up period. Two of these patients (5%) had durable grade 3 toxicity, and one underwent cystoprostatectomy with rectal amputation. Retrospectively, that patient had moderate to severe urinary frequency after the primary radiation course and should have been excluded from this study because of the grade 2-3 RTOG late GU toxicity. The frequency of severe toxicity is somewhat higher than that in the study by Fuller et al^17^ (8%), where periurethral isodose sparing was performed. With the dose constraints to the urethra (D50: 105%) in the study by Fuller et al, the doses to the urethra were similar to those in our study. A possible further periurethral isodose sparing will be challenging in terms of disease control, as local recurrence after radiation therapy often originates close to the urethra.^39,40^ Studies have shown a correlation between dose to the urinary bladder trigone and GU toxicity.^41,42^ With a 5-mm margin around the CTV in our study compared with no margin in the study by Fuller et al,^17^ this part of the bladder may have received a higher dose in our patients. The results from a separate analysis of 28 patients in this study indicated increased GU late grade 2 toxicity in patients with high bladder volume exposure,^27^ emphasizing that reducing doses to the bladder is essential. Compared with surgical salvage treatment, the reported severe toxicity is still low. Severe GU and GI toxicities have been reported in 21% and 1.9% of patients with salvage RP, respectively.^18^

In 2023, the European Society for Radiotherapy and Oncology published recommendations for evidence-based use of ADT combined with EBRT in managing PCa.^43^ The use of additional ADT with EBRT in locally recurrent disease after primary radiation therapy is not mentioned, as randomized trials are missing. The current study used ADT for 6 months either as a luteinizing hormone-releasing hormone analog or antiandrogen, compared with seven of 43 patients treated with ADT in the study by Fuller et al and no use of ADT in the study by Bergamin et al.^17,19^ Patients should be considered for randomized clinical trials to further evaluate the role of additional ADT with EBRT in the salvage situation.

Survival after PSA recurrence following primary radiation therapy for PCa can be prolonged.^44^ The generally favorable prognosis in this group of patients can question whether curative-intent therapy with possible toxic complications is necessary. In this study, more than half of the patients did not experience new recurrence. On the basis of data from studies on recurrence after primary treatment, most of our patients would probably have started treatment with lifelong ADT with adverse effects on quality of life and risk of disease progression.^8,9,45^ This study indicates that re-treatment is meaningful for patients. Larger studies with even longer follow-up are required to detect differences in OS.

Uncertainty of whether the recurrent disease is truly confined to the prostate gland is one of the main reasons why very few patients have received salvage therapy for locally recurrent disease in the past. With the incorporation of prostate-specific membrane antigen positron emission tomography, the probability of detecting a solitary local recurrence at an early stage increases.^46,47^ Improved patient selection urges the need for data to help guide clinical decision making for individual patients with locally recurrent disease. Our study shows that reirradiation with EBRT is a reasonable option for selected patients. Unlike salvage RP and brachytherapy, which require highly specialized centers, reirradiation with EBRT is a technique that most centers that treat patients with radiation therapy can handle in daily practice.

The main strengths of this study are its prospective design and extended follow-up period. Data on baseline demographics at initial diagnosis and salvage were complete, except for one patient with an unknown initial PSA and T stage. Primary treatment was mainly up to date, with dose escalation in most patients and hypofractionation. None of the patients were lost to follow-up.

The small sample size and lack of a control group are limitations of this prospective study. However, there will be difficulties in recruiting patients to a prospective randomized trial, omitting the control arm from the potentially curative treatment traded for lifelong ADT. Staging before salvage treatment is a limitation as only patients with a PSA >10 ng/mL received a bone scan, and few patients had access to 18F-FACBC PET-CT. A standardized biopsy process with systematic and targeted biopsies can optimize cancer detection.^48^ Staging during PSA rise after re-treatment could also be better standardized. Reports on tolerance were based on physician reports rather than patient-reported data. Without this information, the reported toxicity may be underestimated.^49^

This study shows that salvage reirradiation with EBRT for locally recurrent PCa is effective and feasible, with bRFS rates of 81% and 58% at 2 and 5 years, respectively. The durable late GU/GI toxicity was acceptable. One required cystoprostatectomy with rectal amputation, which emphasizes the need to carefully select patients suitable for reirradiation and underlines the potential risk of severe toxicity.
